# Guideline for diagnosis, prophylaxis and treatment of invasive fungal infection post burn injury in China 2013

**DOI:** 10.4103/2321-3868.130182

**Published:** 2014-04-06

**Authors:** Gaoxing Luo, Jianglin Tan, Yizhi Peng, Jun Wu, Yuesheng Huang, Daizhi Peng, Xu Wang, Dahai Hu, Songtao Xie, Guoan Zhang, Chunmao Han, Xiaoyuan Huang, Ciyu Jia, Jiake Chai, Jingning Huan, Guanghua Guo, Jianhua Zhan, Weiguo Xie, Ying Cen, Rong Yu, Huade Chen, Xihua Niu, Yibing Wang, Jinfeng Fu, Baosheng Xue

**Affiliations:** 1 State Key Laboratory of Trauma, Burns and Combined Injury, Institute of Burn Research, Southwest Hospital, The Third Military Medical University, Chongqing, 400038 China; 2 Department of Burns and Cutaneous Surgery, Xijing Hospital, Fourth Military Medical University, Xi’an, Shanxi, China; 3 Department of Burns, Beijing Jishuitan Hospital, Forth Medical College of Peking University, Beijing, China; 4 Department of Burns & Wound Care Center, The Second Affiliated Hospital of Zhejiang University, College of Medicine, Hangzhou, China; 5 Department of Burns and Plastic Surgery, Central South University, Changsha, Hunan, China; 6 Graduate School, Medical College of Chinese PLA, Beijing, China; 7 Department of Burn & Plastic Surgery, The First Affiliated Hospital of PLA General Hospital, Beijing, China; 8 Department of Burn and Plastic Surgery, Ruijin Hospital, School of Medicine, Shanghai Jiao Tong University, Shanghai, China; 9 Department of Critical Care Medicine, The First Affiliated Hospital of Nanchang University, Nanchang, Jiangxi, China; 10 Department of Anesthesia and Critical Care, Union Hospital, Tongji Medical College, Huazhong University of Science and Technology, Wuhan, Hubei, China; 11 Department of Otorhinolaryngology Head and Neck Surgery, West China Hospital, Sichuan University, Chengdu, Sichuan, China; 12 Department of Burns, General Hospital of Guangdong Province, Guangzhou, Guangdong, China; 13 Department of Otolaryngology, Henan Province Hospital, Zhengzhou, Henan, China; 14 Department of Aesthetic Plastic and Burn Surgery, Provincial Hospital Affiliated to Shandong University, Jinan, Shandong, China; 15 Department of Burns, Second Affiliated Hospital of Kunmin Medical University, Yunnan, China; 16 Department of Burns, The First Affiliated Hospital of Chinese Medical University, Shenyang, Liaoning, China

**Keywords:** Burn, injury, guideline, diagnosis, prophylaxis, treatment, invasive fungal infection

## Abstract

Invasive fungal infection is one of the major complication of severe burns which can induce local or systemic inflammatory response and cause serious substantial damage to the patient. The incidence of fungal infection for burn victims is increasing dramatically during recent years. This guideline, organized by Chinese Society of Burn Surgeons, aims to standardize the diagnosis, prevention and treatment of burn invasive fungal infection. It can be used as one of the tools for treatment of major burn patients.

## Introduction

Presently, more and more cases of invasive fungal infections are found post-burn, especially after severe burn injury.[1–3] Factors associated with invasive fungal infection after burn injury include administration of broad-spectrum antibiotics, tracheostomy tubing, mechanical ventilation, parenteral nutrition and invasive monitoring. The increasing rate of nosocomial infections in China may also be another associated factor;[4–6] the increased rate of reporting of fungal infections has also resulted from the development of improved diagnostic techniques for fungal infections and increased awareness among medical staffs.[7,8] The aim of this guideline, developed by Chinese Society of Burn Surgeons, is to standardize the diagnosis, prevention and treatment of invasive fungal infection after burn injury in China. The guideline is based on consensus opinion of 27 doctors and experts from 18 hospitals and burn centers all over China. This version includes 4 parts: The concept, the diagnosis, the prophylaxis and the treatment of burn invasive fungal infection (BIFI).

**Table:** 

Access this article online	
**Quick Response Code**:	**Website**: www.burnstrauma.com
	**DOI**: 10.4103/2321-3868.130182

## Concept of the BIFI

Invasive fungal infection after burn injury is termed as BIFI. BIFI is characterized by local or systemic inflammation, tissue and cell damage caused by colonization, growth and proliferation of various fungi in wounds, blood stream, internal organs, or tissues of burn patients.

## Diagnosis of burn invasive fungal infection

There are 5 clinical features that symbolize BIFI: Host susceptibility, clinical manifestation, fungi detection, histological examination, and imaging and laboratory results.[2,9,10]

### Host susceptibility[12]

- The mechanical barrier severed by intact human skin is destroyed after burn injury, enabling fungi and other microorganisms to invade easily. In addition, necrotic or degenerated tissues are favorable culture medium for colonization and growth of various microorganisms including fungi. Furthermore, immune dysfunction is almost always induced after severe burn injury, with the resultant immunosuppression favoring fungi and other microbial infections.[12–14]
- Patients at increased risk of BIFI include the elderly, children, and the patients with concomitant chronic illness associated with immunosuppression, e.g. diabetes and autoimmune illnesses.[15,16]
- Broad-spectrum antibiotics, commonly administered to patients after severe burns injury, are an additional risk factor for BIFI.[17]
- Severe burn patients combined with inhalation injury, who should always apply tracheostomy tubing and continuous mechanical ventilation for long term.[18]
- Long term use of invasive monitoring, deep venous catheter, and intravenous parenteral nutrition support.[19]
- The severe burn patients always had a long-term stay in hospital.[20,21]
- Other aspects: Compression and moisture of the burn wound and others.[22]

### Clinical manifestations[1,23–25]

#### General condition

- Temperature: Most of the patients suffer continuous fever, usually above 39–#x00B0;C. A few patients manifest remittent fever. Hypothermia is frequently associated with advanced or fulminant infections.
- Consciousness: Systemic infection is associated with altered conscious states, with rapid changes commonly observed. The consciousness alteration can display excitation or inhibitory patterns such as delirium, restlessness, babbing, lethargy, apathy, staring spells, and so forth.
- Respiration system: Dyspnea is commonly observed secondary to the septic process or respiratory infections. The sputum appears gelatinous when the patient has invasive fungal infection in lungs.
- Digestive system: Poor appetite, nausea, anorexia and abdominal distention are common complaints. Dysphagia and odynophagia are common with abnormal bowel actions appearing watery or mucoid. Pediatric patients are often found to have oral mucosal ulcers and pseudo-tunica albuginea.
- Urinary system: The urine may become cloudy. In cases of urethritis, dysuria, frequency and urgency have been observed.
- Circulation: Tacchycardia may be induced by hyperthermia. Arrhythmia may be associated with myocardial infection. Hypotension signifies fulminant disease.
- Others: Microabscesses can occur when fungi disseminate through blood stream to lung, liver, kidney, spleen, brain, blood vessels, eyes and other internal organs.

#### Appearance of the burn wound[26]

- Round or irregularly shaped brown or black mildew or ecthyma gangrenosa can be found on wound crust.
- Wound deepens with bean dregs-like or cheese-like necrosis rapidly and progressively. Infection of mucor often leads to embolism formation with resultant progressive necrosis in muscle or extremities.
- Inflammation around wounds infected with fungi are obvious. Granulation tissue looks gray or bright red but is fragile and bleeds easily. Mucoidal exudate frequently covers the wound. Skin graft appears patent initially but is unable to grow or expand. Hematogenous spread of Aspergillus and Mucor leads to extensive skin hemorrhage or necrosis. Hemorrhagic spots and disseminated erythematous nodules may be found on normal skin.

#### Effect of antibiotics treatment

If presumed to be a bacterial infection and following treatment with antibiotics for 3–5 days, the general symptoms and local appearances do not change or recur with worsening severity.

### Fungus detection

#### Microscopic examination of fungus[27,28]

The diagnosis is established by hyphae found under microscope continuously two times in one kind of sample or in more than two kinds of different samples such as wound exudate, respiratory secretions, urine, or stool. Hyphae may be present in irrigating solution of bronchoalveolar lavage. Pityrosporion ovale cyst, trophont, or intracapsule are found in sputum or bronchoalveolar lavage douche.

#### Culture of fungus[27]

The diagnosis may also be made by a type of fungus isolated and identified from at least 2 samples such as wound exudate, respiratory secretions, urine, blood, and others. The same fungus should be cultured continuously two times from one kind of sample. The fungi are cultured and presented in concentrations of more than 10^6^ and 10^4^ colony forming unit (CFU)/ml in sputum or bronchoalveolar lavage fluid or 10^5^ CFU/ml and 10^5^ CFU/g for urine or stool sample, respectively.

#### Histological evidence[29,31]

Diagnosis by histological evidence for burn wound involves the detection of hyphae in wound biopsy sections, especially fungi around blood vessels, or period acid-schiff (PAS) staining positive in unburnt tissues. The histological diagnosis is the gold standard to burn wound invasive fungal infection.

### Imaging and laboratory examination

- Imaging: Persistence of pulmonary signs of consolidation or new lesions after antibiotics treatment should alert the clinician to the possibility of BIFI. Signs of pulmonary infection with Candida displays as nodular consolidation or/and large consolidation but seldom with cavitation. When infected by Aspergillus, hyperdense consolidation with different shapes can be found under the pleura. Halo signs are usually found around the lesions. A few days later the consolidation field undergoes liquefaction or necrosis, with the formation of hollow shadow or air-crescent signs. Following hematogenous spread, widespread miliary shade can be found disseminating all over the fields.[32–34]
- Blood routine examination: The proportion of neutrophil is below the lower limit of normal value or with sudden descent.[35]
- G test: G test is to detect fungus cell wall ingredient 1,3-β-D glycans by enzyme-linked immunosorbent assay (ELISA). In BIFI, G test displays positive from blood sample for 2 times continuously.[36,37]
- GM test: Galactomannan (GM) assay by ELISA shows optic density is more than 0.8 for 2 times continuously or more than 1.5 in one time from blood or respiratory secretion samples.[38,39]
- Polymerase chain reaction (PCR) test: Specific positive bands are amplified with the specific primers for the fungal 18S transfer ribonucleic acid (tRNA) and ribosomal deoxyribonucleic acid (rDNA).[40,41]

### Diagnostic classification

The diagnosis of BIFI can be divided into 3 grades, i.e. proven diagnosis, probable diagnosis and possible diagnosis.

- Proven diagnosis: Diagnosis of BIFI can be proven once any of the following three terms is met: Histological evidence is present; besides host susceptive factors and clinical manifestation with microorganism identified; besides host susceptive factors and clinical manifestation, with at least 2 imaging or laboratory signs, as described above.
- Probable diagnosis: There is no histological or fungal microorganism evidence. Besides host susceptive factors and clinical manifestation with only one imaging or laboratory test positive.
- Possible diagnosis: Only host susceptibility factors and clinical manifestation are present with no histological, fungal microorganism, or imaging and laboratory examination evidence [Table 1].

**Table 1:** **Diagnostic classification of invasive fungal infection after burn injury**

Classification	Histological evidence	Host susceptive factors	Clinical manifestation	Fungus detection	Imaging and laboratory examination
	+	±	±	±	±
**Proven**	—	+	+	+	±
	—	+	+	—	At least two in five items positive
**Probable**	—	+	+	—	Only one in five items positive
**Possible**	—	+	+	—	—

**+: Positive, ±: Positive or negative, —: No evidence**

Diagnosis of fungal infections of burn wounds from bacterial infection is difficult, mostly reliably made by histological examination of burn wound tissue samples along with culture of the biopsy sample. The bacterial infection can release a large amount of endotoxin into the blood, accompanying the sepsis symptoms. The number of bacteria present around the wound area can be more than 10^5^/g with vasculitis or perivascular inflammation symptoms.

## Prophylaxis of BIFI

### Prophylaxis[42]

Wound should be nursed clean and dry and be allowed to drain completely. Moreover, long-term compression dressing should be avoided. Open wounds should be covered and closed as soon as possible.[24]

Antibiotics should be applied rationally. Broad-spectrum antibiotics should be avoided for long period and focused to culture results as soon as possible.[43]

The use of indwelling catheters should be minimized as far as practicable. If inevitable, these should be nursed carefully. The indwelling time and date should be marked. Invasive arterial and venous lines should be checked and nursed twice a day; the indwelling sites should be changed every 5–7 days for the routine catheter. If the catheter is indwelled on burn wound, the site should be checked and nursed 4 times a day and maintained clean and dry.[44–46]

Tracheal intubation and mechanical ventilation should be avoided and the period should be shortened if possible. The incision site should be cleaned, disinfected, and dressing changed for 1–2 times every day and for 3–4 times a day if it has to go through burn wound. It should be strengthened of respiratory tract management and nursing including sputum aspiration in time, humidification, and other measurements.[24]

Hypoxic ischemic injury of digestive tract should be prevented and treated in time during early stage after burn injury with early enteral feeding and prevention of intestinal flora imbalance. For patients with definite host susceptive factors, ecologic agents such as Bifidobacterium and/or *Bacillus subtilis* are advised to be given from day 3 after burn injury to maintain the intestinal flora balance and inhibit invasive fungal infection from intestinal tract.[47–49]

Metabolic disorders should be treated and corrected in time. Enteral with or without parenteral nutrition support is necessary to meet the need of the metabolism of the severe burn patients.

The treatment of any basic diseases such as diabetes immune hypofunction disease, chronic lung disease, and others should be enhanced. Glucocorticoid and other immunosuppressants that inhibit immune function should be avoided for long term use as far as possible. Immunomodulatory agents should be recommended to be used actively to treat immune dysfunction after severe burn injury. Immune globulin, colony stimulating factor, α-thymopentin, urinary trypsin inhibitor, and others are encouraged.[50,51]

### Empirical antifungal administration[52]

For patients with host susceptive factors, antifungal prophylaxis should be considered.[53,54]

If the patient’s total burn area is more than 50% TBSA, or full thickness burn area is more than 30% TBSA, or moderate to severe inhalation injury is present, or patients suffer from diabetes and other immune-related diseases before burn injury, the elderly empirical antifungals are recommended to start at day 7 after burn injury.

However, we need to face the risks involved in the empirical antifungal therapy. The fungal pathogens have demonstrated increasing degrees of antifungal drug resistance.[55]

Moreover, the burn patients with following conditions should have empirical antifungal therapy[44]: Burn patients that have been applied continuously 2 kinds of broad-spectrum antibiotics for 5 days that cannot be stopped immediately; patients with deep venous or arterial catheter continuously for longer than 2 weeks; patients with trachaeostomy for longer than 2 weeks or mechanical ventilation for longer than 1 week; those administered glucocorticoids or other immunosuppressive agents for longer than 1 week; patients thought necessary to be given empirical pharmacal prevention treatment by doctors.

According to patients’ condition, the course of empirical pharmacal prevention of invasive fungal infection is advised for 1–2 weeks.[53]

Antifungal agents include mainly polyenes (amphotericin B), azoles (fluconazol, itraconazole, voriconazol) and candins (caspofungin and micafungin).[56–59] For prophylaxis purpose, azole or candin agents are always used but not polyenes. Polyenes either amphotericin B or its liposome are the traditional classical antifungal agents, their broad-spectrum antifungal effects are confirmed by many doctors. However, polyenes are used less and less at present for their obvious toxicity and side effects. Flucytosine is another traditional antifungal agents, but because of its nephrotoxicity, it is seldom used alone to treat or prevent fungal infection.

By inhibiting the activity of cytochrome P450-dependent 14-α-lanosterol demethylase, alozes can impact the synthesis of ergosterol, which is the main constituent of fungal cell wall.[60] Candins can destroy the integrin of the fungal cell wall by inhibiting the synthesis of 1-3-β-D polyglucosan, which is one of the most important components of fungi wall. Consequently, the fungi are lysed.[61]

- Pharmacal prevention by oral administration: The agents for oral administration include itraconazole oral solution, itraconazole capsules, fluconazole capsules, vorionazole tablets, and so on.[58,62] The antifungal spectrum of itraconazole covers extensive range of fungi, i.e. Candida, Cryptococcus, Aspergillus, *Blastomyces dermatitidis, Coccidioides immitis*, Paracoccidioides, Sporotrichosis, and others. The preventive dose of itraconazole oral solution or capsule is 200 mg once a day; the first dose should be doubled to 400 mg. To reduce gastrointestinal adverse effects of itraconazole, the solution and capsule can be administered alternately during the first 3 days.[63,64] Fluconazole can prevent almost all kinds of Candida, except *Candida krusei*, invasive infection. The usual preventive dose is 400 mg once a day.[65,66] Voriconazole is sensitive to almost all kinds of Aspergilli, Cryptococcus, Candida including *C. krusei* and *Candida glabrata.* Moreover, vorionazole can prevent Dermatitis Blastomycosis, Coccidioidomycosis Crude Bacteria, Brazilian Cice Coccidioidomycosis, and Histoplasmosis Capsule.[67,68] The common preventive dose of vorionazole is 200 mg, q12 h.
- Prevention by intravenous administration: The agents for intravenous prevention include mainly azoles and candin intravenous formulation.[69,70] The usual preventive dosages of azoles are 200 mg for itraconazole once a day; 4 mg/kg (6 mg/kg for the first loading) for voriconazole, Bid. To prevent the invasive fungal infection, candins are advised 50 mg once a day; the first administration of caspofungin is 70 mg.[71]
- Prevention by oral-intravenous sequential administration: Oral-intravenous sequential administration is recommended to prevent fungal infection. It is advised to give intravenous injection of caspofungin, micafungin, or voriconazole for first 3–5 days for burn patients, then take oral application of itraconazole, fluconazole and vorionazole for 1–2 weeks.

### Monitoring the BIFI

For the severe burn patients at high risk, the clinical signs for invasive fungal infection should be monitored carefully every day, such as body temperature, consciousness, wound appearance, blood routine examination, and so on.[25]

In order to find any microbial evidences for fungal infection in time, microscopic examinations repeatedly are necessary. Various possible samples, i.e. wound secretions, sputum, midstream urine alveolar irrigating solution should be checked promptly under microscope. Meanwhile, above-mentioned and blood samples should be cultured and the identified fungi should be assayed with drug-sensitive test. If possible, biopsy and histological check of the tissues under eschar should be done continuously and repeatedly.[27,28]

For patients with possible infection, chest X-ray or/and computed tomography (CT) should be checked repeatedly to detect pulmonary infections. On the other hand, G test and GM test can be used repeatedly for blood and pleural fluid samples.[32,33]

## Treatment of the BIFI

### Empirical treatment

- Adjust or stop using of broad-spectrum antibiotics: Once the burn patients are suspected of invasive fungal infection, it is necessary to stop using the broad-spectrum antibiotics immediately. If the patient is proven to be complicated with bacterial infection simultaneously, only narrow-spectrum antibiotics can be chosen based on the results of bacteriological and sensitive test.[11,43]
- Intravenous administration of antifungal agents: Vorionazole, 4 mg/kg (6 mg/kg for the first day), q12 h; caspofungin 50 mg once a day (70 mg for the first loading), the infusion time should be not less than 1 h; micafungin, 50 mg (100–150 mg for the first loading) once a day; itraconazole injection, 200 mg, q12 h for the first and second day, then once a day. Two weeks later, it can be adjusted to oral solution, 200 mg once a day.[72,73]

### Wound treatment

Some antifungal agents that are not applied systemically can be used on wound topically to prevent invasive fungal infection. Either 3–5% clotrimazole cream, 10–40% clotrimazole in dimethyl sulfoxide or 100000 U/ml nystatin suspensions can be utilized on wound locally to prevent various fungi colonization, growth, and proliferation. A 1% ketoconazole cream or suspension 2–3 times a day is helpful to prevent Candida or Aspergillus infection on wound.

### Immunotherapy

Immunotherapy agents include granulocyte colony-stimulating factor (G-CSF), granulocyte-macrophage colony-stimulating factor (GM-CSF), macrophage colony-stimulating factor (M-CSF), thymosin, interleukin hormone (interleukins), and infusion of fresh plasma and granulocyte cells. These kinds of agents can improve the host immune function through increasing the numbers and activities of neutrophils and macrophages, which is helpful to prevent and treat the invasive fungal and bacterial infection.[51,74]

### Preemptive treatment

Because severe burn patients are suspected with invasive fungal infection by clinical manifestations, the detection of fungal microorganism and sensitive test should be taken regularly in various samples. Before obtaining the definite results, preemptive treatment is advised to be applied. *Candida tropicalis, Candida parapsilosis*, and so forth are sensitive to azoles and candins agents. Vorionazole, caspofungin, and itraconazole can be chosen for *Blastomyces albicans, C. glabrata*, or *C. krusei* infection. Aspergillus infection is suggested to be treated with vorionazole, the alternative treatment including candin agents, amphotericin B (except of *Aspergillus terreus*), and itraconazole. Amphotericin B is recommended for Zygomycetes (Mucor) infection and combined with surgical treatment is always necessary.[56,57,75,76]

### Targeted treatment

Once results of sensitivities are available, direct and special antifungal agents should be administered intravenously as soon as possible. It is suggested that the antifungal treatment be discontinued after manifestations of invasive fungal infection have disappeared for 2–4 weeks.[53,77,78]

### Surgical treatment

Once invasive fungal infection through burnt tissue is identified, especially Aspergillus and Mucor infection, the infected or suspected tissues should be removed immediately and resolutely. In addition to systemic loading by venous, the sensitive antifungal agents are recommended to be administered around the debrided tissues topically.[24,79] Following complete removal of infected tissue, the wound can be closed by autograft, alloskin, or xenoskin, artificial skin transplantation. Where infected tissue may not be removed, exposure or semi-exposure method of wound closure is recommended with the treated wound kept clean and dry.[80,81]

## Summary

This guideline consisted of four parts including the concept, the diagnosis, the prophylaxis, and the treatment of BIFI. The part of concept defined the BIFI. The part of diagnosis clarified the standards to diagnose BIFI. In the part of prophylaxis, the key points for prevention of BIFI were stated in detail. The treatment of BIFI in the treatment part is expounded form four aspects, i.e. empirical treatment, preemptive treatment, target treatment, and surgical treatment. Generally, the whole guideline supplies with clues and methods, which will help clinician to define, diagnose, prevent, and treat the invasive fungal infection after burn injury.
